# Etiology of Bronchiectasis in the World: Data from the Published National and International Registries

**DOI:** 10.3390/jcm12185782

**Published:** 2023-09-05

**Authors:** Jose Daniel Gómez-Olivas, Grace Oscullo, Miguel Ángel Martínez-García

**Affiliations:** 1 Pneumology Department, Hospital Universitario y Politécnico La Fe, Avenida Fernando Abril Martorell 2026, 46026 Valencia, Spain; jdaniel365@hotmail.com (J.D.G.-O.); graceoscullo@gmail.com (G.O.); 2 CIBERES de Enfermedades Respiratorias, ISCIII, 28029 Madrid, Spain

**Keywords:** bronchiectasis, registry, guidelines, etiology

## Abstract

Bronchiectasis is the third leading chronic inflammatory disease of the airway caused by dozens of pulmonary and extra-pulmonary diseases. Infection by pathogenic microorganisms is very common. We aimed to analyze, for the first time in the literature, the etiology of bronchiectasis throughout the world via data published in national and international registries. A bibliographic search was carried out in PubMed and Web of Science. Seven studies were included, with a total of 27,258 patients from 33 countries of four continents. The most frequent cause of bronchiectasis was post-infectious: 30.5% (range: 19.1–40.4%), followed by idiopathic: 28.7% (18.5–38.1%). Post-tuberculous bronchiectasis accounted for 14.1% (1.8–35.5%), while etiologies associated with COPD and asthma comprised 7% (3.4–10.9%) and 5.2% (2.5–7.8%). In conclusion, there was a high degree of heterogeneity in the relative percentages of the main causes of bronchiectasis in the world, although post-infectious and idiophatic bronchiectasis continue to be the most frequent causes.

## 1. Introduction

A recent consensus of world experts on bronchiectasis defined this disease as a disorder of the airway involving a vicious circle of inflammation, infection and repair that causes dilation of the bronchial lumen, thickening of the bronchial wall and secondary symptoms [1]. These symptoms mostly comprise chronic productive cough (usually with a purulent component) and multiple exacerbations of an infectious profile as well as chronic bronchial infection over the course of the disease’s natural history [2,3,4,5]. The airway inflammation usually observed in bronchiectasis is mixed, with a predominance of neutrophils [6], although some patients may also present an increase in eosinophils [7,8,9]. Bronchiectasis can be caused by underlying pulmonary and extra-pulmonary diseases [10]. Until two decades ago, there were no regulations or patient registries for this disease, but, from the beginning of this century, various scientific societies and research groups began to formulate regulations and create national and international registries of patients with bronchiectasis. Accordingly, our understanding has substantially improved, although it still falls far short of that of other airway diseases such as asthma or chronic obstructive pulmonary disease (COPD). The appearance of these national and international registries, covering thousands of patients, has, among other things, provided more reliable information about the global distribution of the etiologies of bronchiectasis, as well as the associated geographical variability. The objective of this study was to analyze the etiology of bronchiectasis throughout the world via data published in national and international registries.

## 2. Materials and Methods

A descriptive study was carried out via a bibliographical search of articles in PubMed and Web of Science, using the terms “bronchiectasis” (in the title) and “registry” (in the title or abstract). Only those manuscripts that presented etiological data of bronchiectasis from national or international registries were selected, to avoid duplication. Congress communications were excluded. In the case of several studies related to the same registry, the one with the most patients included or with the most recent publication date was chosen. Data from patients with bronchiectasis due to cystic fibrosis were excluded. Since there is a large number of causes known to be capable of generating bronchiectasis, and not all studies make the same division of the different etiological categories, the following etiological groups were considered in order to establish a homogeneous division: (1) idiopathic or etiology unknown, (2) post-infectious, (3) post-tuberculosis, (4) COPD, (5) asthma, (6) primary immunodeficiencies, (7) primary ciliary dyskinesia, (8) systemic disorders (including connective tissue disease, vasculitis and rheumatoid arthritis), (9) allergic bronchopulmonary aspergillosis (ABPA) and (10) non-tuberculous mycobacteria (NTM). The remaining etiologies were included as other causes, depending on the registry (gastro-oesophagic reflux, aspiration, congenital malformations, inflammatory bowel diseases, panbronchiolitis, yellow nail syndrome, Mounier Kuhn, Young syndrome, Swyer–James syndrome, inhalation of gases, inflammatory pneumonitis, bronchiolitis obliterans and alpha 1 antytripsin deficiency).

The values were tabulated as the percentage of the total number of patients analyzed in each of the registries. The global value of all the registries was calculated as the mean percentage (range of values) of patients, according to etiology in those registries that provided data. The chi-square test was used for the comparison of two percentages.

After the bibliographical search, 90 articles were found, related to 11 bronchiectasis registries from Europe (EMBARC) [11], Spain (historical and RIBRON) [12,13], South Korea (KMBARC) [14], India (IBR) [15], the USA (BRR) [16], Australia (ABR) [17], Germany (PROGNOSIS) [18], Italy (IRIDE) [19], the UK (UK-BRONCHUS) [20] and China [21,22]. Manuscripts related to the German [18], Italian [19], Chinese [21] and UK [20] registries were excluded, as they did not present etiological data nor did they consist of a methodological description of the registry.

## 3. Results

Seven studies were included, with a total of 27,258 patients with bronchiectasis from 33 countries with a mean data inclusion period of 5.6 years. Of these, 16,963 patients came from the European registry (62.2%) covering 28 countries [11]. In global terms, the most frequent prevalence of bronchiectasis was post-infectious: 30.5% (range: 19.1–40.4%), followed by idiopathic bronchiectasis: 28.7% (18.5–38.1%). Post-tuberculous bronchiectasis accounted for 14.1% (1.8–35.5%), while etiologies associated with COPD and asthma comprised 7% (3.4–10.9%) and 5.2% (2.5–7.8%), respectively (COPD and asthma were not included in the data from the American registry [16], since it considered them comorbidities rather than possible causes, and nor did it provide data related to post-infectious or idiopathic bronchiectasis). Other etiologies did not account for more than 10% of the causes in any of the registries analyzed, as can be seen in Table 1. It is worth noting that in the study relating to the European EMBARC registry [11], with data from 28 countries, the most common etiology was idiopathic versus infectious (38% vs. 21.2%) in all areas apart from Central and Eastern Europe, where it was post-infectious versus idiopathic etiology (30% vs. 26.4%).

Finally, a comparison of etiological percentages over time was only possible with the Spanish registries, since there were data from two sources: the historical registry [12] with data from 2047 patients included between 2002 and 2011, and the computerized registry (RIBRON) with data from 2615 patients included between 2015 and 2023 [13,23]. When comparing these two registries, a significant decrease in idiopathic (24.2% vs. 18.5%) and post-tuberculous (18.6% vs. 13.5%) bronchiectasis and a significant increase in post-infectious bronchiectasis (30% vs. 40.4%) and bronchiectasis due to COPD (7.8% vs. 10.9%) and asthma (5.4% vs. 7.8%%) were observed; all of these changes presented a *p* < 0.001.

## 4. Discussion

The following scientific letter shows, for the first time, the distribution of bronchiectasis etiologies worldwide, based on national and international registries comprising data from more than 27,000 patients. Perhaps the most striking characteristic is the great heterogeneity in the percentage of the most frequent causes of bronchiectasis in the world. The post-infectious cause continues to be the most frequent in most territories and even tends to increase in some of them. The post-tuberculosis cause presents the greatest variation, with a range between 1.8% and 35%. This percentage is directly related to the participating countries’ socio-sanitary characteristics, so, while in Europe [11], the USA [16] and Australia [17], it did not exceed 5% on average, it exceeded 35% in India [15]. Data from large series of patients with bronchiectasis from Latin American countries [24] and some areas of China [22,25,26] showed percentages similar to those of India [15]. Similarly, when the European registry [11] was analyzed in detail, it could be seen that, although in global terms the percentage of post-tuberculosis bronchiectasis stood at 4.9%, this percentage ranged from 2.9% in the UK and 3.1% in Western and Northern Europe to 8.5% and 10.8% in Southern and Central/Eastern Europe, respectively, i.e., there was a clear gradient of a higher prevalence of post-tuberculosis bronchiectasis in those countries on the eastern and southern fringes of Europe. Finally, a significant decrease was observed in such cases in Spain, after 20 years of evolution from 18.6% to 13.5% [12,13]. These findings may also be related to changes in socio-sanitary conditions, since it must be taken into account that several decades may pass between the diagnosis of pulmonary tuberculosis and the appearance of clinically relevant secondary bronchiectasis secondary to tuberculous infection.

However, a large percentage of patients still presented no known etiology (idiopathic) (28.7%), with this percentage rising to over 35% in many European countries [11] and in South Korea [14], but it is striking that the percentage of idiopathic bronchiectasis was low in India (21.4%) [15]. The same trend was observed when analyzing large series of patients from Latin American countries (26%) [24] and Eastern European countries such as Croatia, Slovenia, Bulgaria and North Macedonia (less than 10%) [11]. This finding can probably be explained by the high percentage of post-infectious and post-tuberculous bronchiectasis existing in these countries. At this point, it is important to note that there may be an overdiagnosis of post-infectious bronchiectasis, especially when attributed to long-past or childhood infections, since this diagnosis is usually reached via a process of elimination. This phenomenon cannot be ruled out as an explanation for the significant reduction (18.6% to 13.5%) in idiopathic bronchiectasis observed in Spain [12,13]. In any case, the percentage of idiopathic bronchiectasis is also likely to decrease over time as a result of the greater number and quality of existing bronchiectasis regulations and the health community’s growing awareness of the importance of a good etiological diagnosis, especially in a country like Spain that has had bronchiectasis registries and regulations in place for almost two decades.

Finally, the percentage of bronchiectasis due to COPD or asthma is noteworthy, and it has possibly increased over time, as shown by the Spanish data [12,13]. Although there are no data showing a clear causal relationship with these two diseases, an increase in the studies on the relationship between bronchiectasis and COPD or asthma has led to a greater awareness on the part of health professionals.

The strengths of this study included, above all, that it was the first to be carried out with data from the world’s main national and international registries [11,12,13,14,15,16,17], with more than 27,000 individuals included, thereby establishing a global vision of the most frequent etiologies of bronchiectasis in four continents and 33 countries. Also, thanks to data from Spanish registries [12,13] with more than 4600 patients included, it was possible to observe the evolution of some etiologies of bronchiectasis, although these data cannot be extrapolated to other countries. The study’s limitations included the fact that direct data were not available from registries in some very important areas of the world, such as China or South America, although it was possible to recover some etiological data of interest from large series of patients published in these regions. Lastly, it should be noted that the manuscripts concerning the US registry [16] did not present complete etiological information but focused on related diseases or comorbidities that cannot always be considered true causes of bronchiectasis. Furthermore, this registry was dominated by centers specializing in the study of non-tuberculous mycobacteria (NTM), as evidenced by the fact that 63% of the patients included therein presented an isolation of an NTM, compared to only 1% of the patients in the European registry.

## 5. Conclusions

In conclusion, there is a high degree of heterogeneity in the relative percentages of the main causes of bronchiectasis in the world, although post-infectious bronchiectasis continues to be the most frequent cause, the percentage of idiopathic bronchiectasis remains high and there has been an increase in the percentage of other causes such as COPD or asthma. A consensus of world experts on an appropriate protocol is still needed to reduce as much as possible the percentage of idiopathic bronchiectasis, especially in those etiologies that could be potentially treatable [27,28,29,30,31].

## Figures and Tables

**Table 1:** Most frequent etiologies of bronchiectasis in national and international registries.

Etiology	EMBARC Europe	Historical Spain	RIBRON Spain	BRR * USA	IBR India	ABR Australia	KMBARC South Korea
Number	16,963	2047	2615	1826	2195	566	931
Period of inclusion	2015–2022	2002–2011	2015–2023	2008–2014	2015–2017	2016–2018	2018–2021
ETIOLOGY							
Idiopathic	38.1%	24.2%	18.5%	NA	21.4%	32.5%	37.2%
PI	21.6%	30%	40.4%	NA	22.4%	28.1%	19.1%
Post-TB	4.9%	18.6%	13.5%	4%	35.5%	1.8%	20.1%
COPD	8.1%	7.8%	10.9%	20%	5.3%	3.4%	6.4%
Asthma	6.9%	5.4%	7.8%	29%	2.5%	3.7%	5%
Primary ID	4.1%	9.4%	4.2%	5%	1%	3.7%	NA
PCD	3%	2.9%	4.2%	3%	<1%	3.9%	NA
Systemic disorders	4.1%	1.4%	4%	NA	1.8%	2.8%	17
ABPA	2.8%	0.9%	0.9%	NA	8.9%	3.9%	NA **
NTM	1%	3%	4%	63%	1.8%	8.5%	4.6
Others	5.4%	15%	3%	NA	0%	5.3%	2.8%

* The American registry (BRR-US) refers to related diseases rather than etiologies. ** In the case of the KMBARC registry in South Korea, bronchiectasis due to ABPA was included under “others”. PI: Post-infectious; Post-TB: Post-tuberculosis; COPD: Chronic Obstructive Pulmonary disease; ID: Immunodeficiency; PCD: Primary Ciliar dyskinesia; ABPA: Allergic Bronchopulmonary Aspergillosis; NTM: Non-tuberculous mucobacteria.

## Data Availability

All data used are already published.
